# Phenomenology yesterday, today, and tomorrow: a proposed phenomenological response to the double challenges of contemporary recovery-oriented person-centered mental health care

**DOI:** 10.3389/fpsyg.2023.1240095

**Published:** 2023-09-21

**Authors:** Guilherme Messas, Giovanni Stanghellini, K. W. M. (Bill) Fulford

**Affiliations:** ^1^Department of Mental Health, Santa Casa de Sao Paulo School of Medical Sciences, São Paulo, Brazil; ^2^The Collaborating Centre for Values-Based Practice, St Catherine’s College, Oxford, United Kingdom; ^3^Department of Health Sciences, University of Florence, Florence, Italy; ^4^‘D. Portales’ University, Santiago, Chile; ^5^St Catherine’s College, Faculty of Philosophy, University of Oxford, Oxford, United Kingdom; ^6^Medical School, University of Warwick, Coventry, United Kingdom

**Keywords:** phenomenological psychopathology, history of psychiatry, person-centered care, recovery, contemporary psychiatry, values-based practice

## Abstract

This paper argues that a dialectical synthesis of phenomenology’s traditional twin roles in psychiatry (one science-centered, the other individual-centered) is needed to support the recovery-oriented practice that is at the heart of contemporary person-centered mental health care. The paper is in two main sections. Section I illustrates the different ways in which phenomenology’s two roles have played out over three significant periods of the history of phenomenology in 20th century psychiatry: with the introduction of phenomenology in Karl Jaspers’ General Psychopathology in 1913; with the development a few years later of structural phenomenological psychopathology; and in the period of post-War humanism. Section II is concerned with the role of phenomenology in contemporary mental health. There has been a turn to phenomenology in the current period, we argue, in response to what amounts to an uncoupling of academic psychiatry from front-line clinical care. Corresponding with the two roles of phenomenology, this uncoupling has both scientific aspects and clinical aspects. The latter, we suggest, is most fully expressed in a new model of “recovery,” defined, not by the values of professionals as experts-by-training, but by the values of patients and carers as experts-by-experience, specifically, by what is important to the quality of life of the individual concerned in the situation in question. We illustrate the importance of recovery, so defined, and the challenges raised by it for both the evidence-base and the values-base of clinical decision-making, with brief clinical vignettes. It is to these challenges we argue, that phenomenology through a synthesis of its twin roles is uniquely equipped to respond. Noting, however, the many barriers to such a synthesis, we argue that in the current state of development of the field, it is by way of a dialectical synthesis of phenomenology’s roles that we should proceed. From such a dialectic, a genuine synthesis of roles may ultimately emerge. We conclude with a note on the wider significance of these developments, arguing that contrary to 20th century stereotypes, they show psychiatry to be leading the way for healthcare as a whole, in developing the resources for 21st century person-centered clinical care.

In this article we take the long view, arguing that there is a key lesson to be drawn from the history of phenomenology in 20th century Western psychiatry for its role today in the 21st century. The parallels between the two periods have been have been widely recited: preceding both has been a flowering of objective brain sciences; yet both have been marked by crises of disappointment at the failures of their respective brain sciences to deliver improvements in front-line clinical care; and both have witnessed a turn to phenomenology as a crisis response. This as we indicate below, is perhaps most clearly shown in Karl Jaspers’ introduction of phenomenology to psychiatry in his 1913 General Psychopathology. But later turns to phenomenology, as we illustrate below with two further periods in 20th century phenomenological psychiatry, although in part, like Jaspers, science-centered (with phenomenology focused on enhancing the role of empirical science in psychiatry), have been in part individual-centered as well (with phenomenology focused on enhancing the role of humanism in psychiatry).

There are of course also differences between the two centuries: in the range of disorders treated by mental health services—yesterday, largely confined to severe conditions such as severe psychoses like schizophrenia and manic-depressive psychoses, today extending to milder conditions marked in particular by anxiety and depression and to the “psychopathology of the present,” including, e.g., addictions and so called eating disorders and borderline personality disorders. Second, in the location of care—yesterday, predominantly within closed institutions, today, though still predominantly within closed institutions worldwide, with a tendency, based on World Health Organization (2022) guidelines, toward open and community-based. But the key difference, the difference that we argue in this article is key to the role of phenomenology today, is the enhanced importance attached in contemporary clinical care to the voices of patients and carers as experts-by-experience, alongside those of researchers and clinicians as experts-by-training.

We discuss this feature of contemporary mental health care in more detail later in the paper. At its heart, we suggest, and unifying its other manifestations, is a novel concept of “recovery,” a concept defined, not by the reduction or abolition of symptoms (reflecting the aims of researchers and clinicians) but rather by the restoration of a good quality of life as defined by the values of (by what matters or is important to) the individual concerned (recovery of this kind thus may, or it may not, include symptom control). Although increasingly widely acknowledged as underpinning best practice in contemporary person-centered mental health care (Slade et al., 2014), recovery, so defined, represents a double challenge for psychiatry in its provision of care. It requires of psychiatry, care that is responsive, on the one hand, to the unique features (including the unique values) of the individual, and on the other hand, to the generalizable evidence derived from empirical medical science. This is why, as we set out toward the end of the paper, we propose that a synthesis of phenomenology’s twin roles, its individual-centered and science-centered roles, would make it uniquely well-fitted to respond to the corresponding twin challenges posed by contemporary recovery-oriented mental health care.

The paper is in two main sections. Section 1 reviews the history of phenomenology in the 20th century, illustrating its twin roles at three critical junctures: at its introduction with Karl Jaspers in the early 20th century; with the development a few years later of structural phenomenological psychopathology; and during the period of post-War humanism. Our “take” on this history is that phenomenology over this period can be seen to have been caught between the two roles noted above, one science-centered, the other individual-centered (we define these roles further by illustration in section 1). Section 2 then turns to the contemporary period with an outline of the emergence of recovery-oriented mental health care and the potential role of phenomenology as a response to the twin challenges presented by it. Our proposal, again, is that a synthesis (we specify a dialectical synthesis) of phenomenology’s twin roles would make it uniquely well placed to support the contemporary crisis of psychiatry–a synthesis serving the twin challenges presented by the model of recovery that sits at the heart of contemporary person-centered mental health care.

## 1. History

In this section, we explore through exemplar periods, two roles taken by phenomenology in 20th century psychiatry: we call these, respectively, its science-centered role (employed in supporting psychiatry to engage with the generalizable evidence provided by empirical science) and its individual-centered role (employed in supporting psychiatry to engage humanistically with its patients as individually unique human beings). As we will see, these two roles, as played out in different ways through three periods of 20th century psychiatry, were not always sharply distinct.

### 1.1. Karl Jaspers, Kurt Schneider, and descriptive psychopathology

Applying the phenomenological method to the classification of abnormal mental states, Karl Jaspers, building on his earlier work (Jaspers, 1912/1968), published *General Psychopathology* in 1913 (Jaspers, 1913/1997). In this, reflecting the science-centered role of phenomenology, he created a language and a system–“descriptive psychopathology”–whose categories were meant to rigorously define and mark out the pathological modifications of human experiences with which psychiatry as a medical discipline is concerned. Troubled as Jaspers was by what he perceived as the hubristic claims of the brain sciences of his day (he was well-placed to criticize, having worked as a researcher in Franz Nissl’s brain science laboratory), Jaspers aimed to endow psychiatry with a bulwark of rigorous understanding of abnormal mental phenomena in order to bring it within the arena of medical science.

He recognized that the epistemological foundations of this project should stem from the field of real, knowable phenomena, clearly distinguishable via precise and valid methods; and that these phenomena needed to fit into a systematic and explicit conceptual framework. As he put it “Psychopathology is concerned with every psychic reality which we can render intelligible by a concept of constant significance” (Jaspers, 1913/1997, p. 2). It was to achieve all this that Jaspers, again reflecting the science-centered role of phenomenology, based his descriptive psychopathology on phenomenology which, he considered, offered a *science of experience*. Jaspersian phenomenology thus aims to describe the manner in which experiences appear to consciousness. Its goal is to find the essential *laws* of consciousness, i.e., how phenomena present themselves to consciousness and how they are organized within it. The categories of his descriptive psychopathology, were, in Jaspers’ view, the uniform, unchanging “boxes” into which the pathological modifications of the human experience could be placed so that they could be recognized and studied scientifically.

The science-centered role of phenomenology continues with Kurt Schneider who built on Jaspers’ work to create a bridge between descriptive psychopathology and nosography, the discipline which researches the signs and symptoms useful for the diagnostic classification of mental diseases (Schneider, 1959). Starting with Kraepelin’s nosology with its subdivision of madness into two opposing categories, dementia praecox (later called schizophrenia) versus the manic-depressive insanity, Schneider adopted the method of Jaspersian descriptive psychopathology to create a “driving belt” between nosography and psychopathology. This allowed him to establish clear (although somewhat arbitrary) criteria for diagnoses, based on psychopathological categories.

Schneider’s epistemology, building on Jaspers’ phenomenological psychopathology, thus represented a determined attempt by psychiatry–on a par with the neuroscientific turn in the late nineteenth century–to be admitted into the ranks of medical disciplines. But in this, while indeed reflecting the operation of phenomenology’s science-centered role, it does so at the expense of its role in understanding the individual. For generations of psychiatrists, Schneiderian clinical psychopathology has made psychopathology ancillary to nosography–that is to say, clinical psychopathology has subordinated the task of understanding the being-in-the-world of individuals affected by mental disorders, to that of assigning them a diagnostic label.

A clear example of this is provided by so-called “Schneiderian first rank symptoms”–including delusional perceptions, verbal-acoustic hallucinations and experiences of passive control. The way Schneider’s first rank symptom are used diagnostically by psychiatrists, leaves little or no space for trying to understand *what it is like* to experience a delusional perception or a verbal-acoustic hallucination or passive control, since *all the emphasis is put on their diagnostic and nosographic significance*. Psychopathology has thus ceased to be concerned with understanding the feeling and meaning of abnormal human phenomena; it has become instead the science of the symptoms of mental illnesses.

This is not as such to critique Schneider’s attempt to define as clearly as possible the characteristics of psychotic symptoms. This was after all, a serious-minded attempt to counter the diagnostic free-for-all of the time, an attempt that led some years later to the development of our contemporary descriptive ICD and DSM classifications (Kendell, 1975). Our point is that, when reduced merely to an ancillary position in relation to nosographic diagnosis, phenomenological psychopathology is at risk of giving importance to symptoms merely as indexes of an underlying illness, rather than to understanding the essential subjective features of the lived experience of abnormal mental phenomena. Psychopathological phenomena should not in our view, be understood merely as expressions of a disease process, but rather as relational phenomena which materialize and become meaningful in the historical context of an individual’s life-history.

So understood, therefor, phenomenological psychopathology may have lost sight of what, reflecting the twin roles of phenomenology described above, one of us has called elsewhere its “centaurical” (or two-way) nature (Stanghellini, 2005), that is as a discipline aimed at both *describing/classifying* and *understanding* mental health issues. This is clear in Jaspers’ work leading up to General Psychopathology: it is made explicit for example in the title of a key paper from this period–‘Causal *and meaningful* connexions between life history and psychosis’ (Jaspers, 1913, emphasis added). In contemporary Schneider-derived psychopathology, therefor, phenomenology has given up its own Jaspersian mandate: viz., elaborating conceptual tools which would allow a rigorous comprehension of the pathological phenomena embedded in a person’s subjectivity. Small wonder, then, that recycled by mainstream psychiatry, phenomenology has become a discipline that merely picks out those symptoms which, operationally defined and thereby reduced to their trivial meanings (Rossi Monti and Stanghellini, 1996), are considered useful to (because reliably identifiable in the context of) diagnostic procedures. Consider, for example, the operational definition of “hallucination” as “a sensory perception that has the compelling sense of reality of a true perception without external stimulation of the relevant sensory organ”; or of “delusions” as “false beliefs based on incorrect inference about external reality.” For centuries philosophers have been struggling with such concepts as “sense of reality,” “external reality,” “belief,” “inference,” as well as many others. The accepted psychiatric definitions ignore what they perceive as the irrelevant metaphysical musings of philosophy, relying instead on vague, ill-defined and even metaphorical slogans. We note the philosophical resources potentially supporting phenomenology in responding to the challenges of contemporary psychiatry toward the end of this paper.

### 1.2. Structural phenomenology and the “Vision of Man”

So deeply embedded is the science-centered role of phenomenology in mainstream psychiatry that the Jaspers/Schneider history has appeared in world-class journals as an origin-story for contemporary psychiatry (see, e.g., Huber, 2002). Yet in General Psychopathology, Jaspers himself emphasizes that the phenomenology on which his book was based is “only one point of view among many and holds a *subordinate position* at that” (Jaspers, 1913/1997, p. 48, emphasis added). History has shown him to be right. Indeed in 1922, within less than 10 years of the publication of General Psychopathology, a whole new movement in phenomenological psychopathology was launched, a “phenomenological psychopathology” as it came to be called, “in the strict sense” (Tatossian, 1978/2002). It is indeed this phenomenology, that, through the work of a distinguished series of structuralist/hermeneutic phenomenologists–including Binswanger, Minkowski, Straus, Fischer, Wyrsch, Rümke, Maldiney, Kuhn, Kimura, Boss, Blankenburg, van den Berg, von Bayer, Zutt, Tellenbach, Binder, Von Gebsatell and others–is regarded by many as the true foundation of contemporary phenomenological psychopathology.

The crucial point that distinguished this new phenomenology from that envisaged by Jaspers was the introduction of the notion of structure (Stanghellini, 2009; hence, we call this in what follows “structural psychopathology”). The structural version of phenomenology probes the experiential field beyond the first-person narrative, revealing the pre-reflexive (or transcendental) structures of abnormal experience. Such pre-reflexive structures, although by definition not accessible by the mere description of the first-person experiences, can be assessed both by a reflective endeavor of the experiencing person and/or by the second-person hermeneutic endeavors of an expert directly engaged with and attentive to the accounts of the individual’s life world (Messas et al., 2022).

So described, structural phenomenology reflects the individual-centered role of phenomenology in psychiatry. Indeed, Ludwig Binswanger’s seminal anthropological work (for example in Binswanger, 1957) was conceived as a foundation for structural phenomenology; and Jaspers himself, in the work noted above leading to General Psychopathology, drew in effect on the individual-centered role in turning to the work of Max Weber and others on the significance of meaningful understanding as the goal of what at the time were called the “human sciences” (these included, e.g., history, so we might perhaps call them today the “humanities”).

Yet, just as the individual-centered role of phenomenology is evident in Jaspers’ ostensibly science-centered use of phenomenology, so the science-centered role of phenomenology is evident in the ostensibly individual-centered structural phenomenology. For, as the philosopher of psychopathology, Basso (2012) has argued, the founders of structural phenomenology were responding, like Jaspers, to needs directly dictated by the dominant scientific paradigm. It is quite true that Binswanger, above all, developed his elaborate critique of the conception of humankind proposed by the natural sciences and even psychoanalysis, as a way of seeking to distance himself from both. However, the focus of his psychopathological interests never swerved from another classic question of psychiatry: the issue of *diagnosis*. As Basso points out, the founding fathers of structural phenomenological psychopathology engaged in an ongoing conflict with their contemporaries over the nature of diagnosis and, as a consequence, how best to access it.

There was, however, a major conceptual innovation in the approach of phenomenological psychopathologists to diagnosis which gives us grounds to see it as a partial and individual-centered break with the dominant science-centered paradigm. Thus, the classic structural phenomenologists shifted the logic of understanding mental disorders from their external causes to a search for a form of internal *logic*, mediated by the notion of structure. Structural psychopathology builds in this regard on the studies of Minkowski, Straus, Binswanger, Ey, Tellenbach, and Blankenburg, among many others. Danish linguist Hjelmslev (1971) succinctly defines a structure in this tradition as “an autonomous entity of internal dependences” (p. 28). To display a structure thus means displaying how the parts stand in a relationship to each other of reciprocal meaningful expression (Stanghellini, 2009). Structural psychopathology assumes that the manifold phenomena of a given mental disorder have a *meaningful* coherence. Rather than being a mere aggregate of symptoms, they form a structure, i.e., a *meaningful* whole. Clinicians can thus find, and should look for, internal links of meaning between the various aspects of a person’s *individual* experience. The method of phenomenological psychopathology is a prerequisite for moving beyond pure static description of the single experiences of the patient toward the illumination of the structures of subjectivity that generate and shape the phenomenal world–the patient’s *life-world*. This approach can provide the background for unfolding the manifold of the patient’s experiences, including all those details that resist standard semiological classification; and it can uncover the architectural nexus that lends coherence and continuity to them within the individual’s subjectivity.

By introducing the idea of structure, investigations of the reasons for altered mental states could thus be sought in the very concatenation and coherence of individual experience. This shift in emphasis had a direct epistemological impact on the perception of scientific objectivity. Schizophrenia, for example, should no longer be investigated objectively as an alteration of neurobiological functions, but as a breakdown of the constituent structures of individual reality – of the implicit and pre-reflective “functions” which constitute the individual’s experience of reality. It is evident that this new internal, structural objectivity attributed to experience, making it relatively independent of brain findings, lent the science of psychology new significance vis-a-vis the positivist paradigm. If the object of science was a person’s individual experience, then the science of psychology–in tandem with its psychopathological dimension–was hierarchically superior to all other sciences of the mind. This, the first incursion of humanism at the forefront of a scientific agenda in the 20th century, was rooted in the then new understanding that the mind should be the core object of psychiatric enquiry.

The awkward and thorny question of diagnosis in psychiatry continued to be the focus of attention of scientific endeavors (Kendell, 1975). This revealed how the classical authors in structural psychopathology always deeply identified with the psychiatry of their time, criticizing only its lack of objectivity, which they attributed to the failings of positivism. If they needed to build a new view of the human, often drawing on philosophical language for their psychopathological conceptions, they did so to give voice to the strict needs of psychiatry itself (Basso, 2009). Their philosophical output, in the form of anthropological reflections, served the pragmatic purpose of lending the science and practice of psychiatry greater objectivity. The association the founding fathers of structural psychopathology felt with the great themes and procedures of psychiatry can be seen, for example, in the organic coexistence of philosophically inspired psychopathological ideas with radically biological therapeutic conduct, such as for instance antidepressant medications (Kuhn, 1958) and electroconvulsive therapy (Tellenbach, 1983). This is an association that may seem scientifically spurious or even morally corrupt in the eyes of many advocates of phenomenology today. But the novel notion of structure granted a status of reality to the object of psychiatry that it had enjoyed at no previous period in the history of the discipline.

This new objectivity–only partially incorporated into psychiatry–should be understood within the context in which it emerged. Although it provided a conceptual framework that enabled a renewal of the psychiatric agenda, its social influence was very limited. We must keep in mind as we noted earlier, that, in the period in which it was born and flourished, psychiatry as a social institution was limited to addressing what today would be referred to as severe mental disorders, mostly of a psychotic nature; and it did so within hospital institutions with a predominantly asylum-like profile. During this period, psychiatry, in its mainstream orientation, was therefore restricted to hospitals and to the suffering of severe patients. In its ideological dimension, however, it can be said that the positivism of psychiatry in the late 19th century and early 20th century was guided by a perspective whose humanistic foundation was the aim of benefitting the human race. Mental illness was seen mostly as a deviation from this goal, something that should be corrected by the scientific identification of the mechanics behind its production in individuals, or, in its more radical and deleterious expression, banished from society through social hygiene policies (Luty, 2014).

The humanist reaction of phenomenology offered an alternative approach to biological positivism of this variety, creating a view of human beings that survives to this day as a counter-current to positivist ideologies. As such, it contributes to anti-stereotypical thinking, conceiving vulnerability to mental disorders to be an intrinsic property of being human: persons affected by mental symptoms are, on this view, closer than “normals” to the core of the human condition (Binswanger, 1933). From this perspective, every question about psychopathological symptoms can assume the form of an interrogation of their meanings; and the way we answer such questions can inform us about the core and defining features of human existence. Thus, anticipating our proposal in section II of this paper, structural thinking so described, can be the source of a new medical, anthropological, technological, social and political understanding of psychopathology (Stanghellini, in press). Such an understanding, as we will see, is well tuned to the challenges of contemporary recovery-oriented mental health care.

However, in the period when it was first developed, structural thinking did not have sufficient strength to influence mainstream psychiatry. The limited importance attached to it can be seen, for example, in the marginal position of the founding fathers of structural phenomenological psychopathology: Binswanger pursued his whole career in his private clinic; Minkowski never held an academic position; and Straus, despite earning some academic prestige in the American Midwest after migrating to the USA, never actually influenced the great debates about psychiatry in that country. Similarly, none of the first-generation proponents of the whole Italian tradition of phenomenological psychopathology were appointed to important positions in their local universities.

Before moving on, it is worth noting one further characteristic of this stage in the development of phenomenological psychopathology that may have reduced its influence. This was its tendency to engage extensively with its philosophical foundations, thus presenting psychopathology as an arcane field of hermetic discussion with little relevance to the working psychiatrist. The excessive use of philosophical jargon in psychopathology (e.g., Dasein, being-in-the world; Befindlichkeit) certainly did nothing to help ease the way for phenomenology into mainstream psychiatry. The rare exceptions in the period concerned a conception of psychotherapy mostly influenced by the psychoanalytical worldview, in which the tenets of phenomenology were only incorporated into care superficially (Toepfer, 2013).

This disconnection from clinical care is significant not least as an indication of the importance of maintaining *both* of phenomenology’s roles in psychiatry. There are similar indications later in the century, with literature, building on the late work of Jaspers (1959) and the philosopher Martin Heidegger, marked by a rejection of technology as an obstacle to humanism and thus blocking the potentially fertile use particularly of psychopharmacology in psychiatry. These limitations indeed extend to person-centered care since, as recent empirical work has shown, while some patients reject, many others positively value medical diagnosis and intervention (Colombo et al., 2003): hence any genuinely person-centered model of mental health care must accommodate both. The need for both roles of phenomenology is evident in a different way (i.e., by default) in our third exemplar period, that of post-war humanism.

### 1.3. Post-World-War-II humanism and phenomenology in psychiatry

After the horrors of World War II, society determined to rebuild civilization based on humanistic values. In the context of this humanistic renewal, mental health came to be understood in person-centered terms as a right of minoritized, underprivileged and vulnerable people, with mental health issues escaping the limits of hospital institutions and gaining a position in society as a whole. Psychiatry, now transformed into “mental health,” expanded into a range of different independent professions (later to be organized as multidisciplinary mental health teams) and set about adapting itself to meet the requirements of the new paradigm.

Not surprisingly, phenomenological humanism had a prominent position in this new person-centered agenda. The interest in diagnosis, having flourished in the preceding period and continuing to occupy the attention of mainstream psychiatry (Fulford and Sartorius, 2009), seemed to the humanistic reformers of the time too bound to the positivist scientific worldview. As phenomenology was increasingly less called on to explore the scientific foundations of diagnosis, its influence grew in reflections on the existential meanings of mental disorders and, particularly for some prominent phenomenology-influenced authors, the role of society in the genesis of mental health issues.

Phenomenology participated in this new current of thinking in two streams, which we will call the social and the individual. The social stream of post-war psychiatry appeared in its most conspicuous form as a deconstructionist trend of the institutional establishments of mainstream psychiatry. Led by figures such as R.D. Laing in the UK, David Cooper, born in South Africa but working in a number of countries (who coined the term “anti-psychiatry”) and Franco Basaglia in Italy, and influenced by the work of the French philosopher and historian, Michel Foucault, this social stream of phenomenologically-influenced reformers, directed their attention over a period running from about 1960 to 1980, toward a critical deconstruction of the function of psychiatry as a whole in society.

Firmly refuting the previous paradigm, this stream of post-war anti-psychiatry phenomenology identified the old psychiatric institutions as tokens of its failings, and made institutional transformation a lynchpin of the global renewal that it sought. In support of this position, it aimed to demonstrate how the diagnoses by which the previous paradigm was characterized, were responsible for one of the heinous policies in the recent history of the period, namely, eugenics and social stigma.

So understood, the shifting of the critical focus from psychopathology toward the psychiatric institution was a product of an understanding of psychopathology as a science that was tainted by ideological and normative tenets. As a consequence, psychopathology, including Jaspersian-Schneiderian phenomenological psychopathology, came to be seen as a science subordinated to sociology and politics. It will be evident that within this scenario, the classic debates on the objectivity of diagnosis had to be left behind, as it was the very objectivity of mental disorder that was *sub judice*. Thus, shunning the diagnostic traditions of psychiatry, the social stream of phenomenologically-influenced reformers opened a gap between the philosophical humanistic conception of psychiatry and the issues of diagnosis, a gap that proved, ultimately, a barrier to the power of phenomenology to influence the care of severe mental disorders.

Parallel to these developments, as already indicated, was an individual stream of humanistic phenomenology: *phenomenology as humanist psychology*. This current, in contrast with the previous structuralist tradition in phenomenology, rejected diagnosis in favor of philosophical humanism, conceiving phenomenology as a field of knowledge of a largely existential kind (May et al., 1958) and drawing inspiration in particular from psychotherapy. Through this individualist construction of phenomenology, authors such as Medard Boss, Carl Rogers, Rollo May, and Erich Fromm, delved into the totality of existence and its dilemmas, seeking to understand mental disorders in a way that deconstructed the classic themes of psychiatry, particularly the role of biological factors in both the causes and the treatment of mental disorders.

These developments inspired a psychology that was closer to a kind of philosophical counseling than a technical discipline. As such, it was criticized for its individualistic approach (Sass, 1989) and failure to strictly observe the principle of existence as contextually positioned. In this terrain of counseling about the deep dilemmas of life [“cf. Jaspers’ limit situations” (Fuchs, 2013)], there was also little room left for the science-centered agenda of the earlier phenomenological movement, based on a search for objectivity. The philosophical prism of the existentialist current in effect bypassed the problem of science completely, since it took existence to be alien to the scientific endeavor. As such, phenomenological psychotherapy inspired by existentialism emerged as the most persuasive approach to transforming the mental health mainstream, aligning itself in the process with the anti-technology (“conservative”) aspects of the social stream.

In the anti-scientific atmosphere of a period marked (justifiably perhaps) by compassionate humanitarianism, there was a consensus that it was more appropriate to approach mental suffering through social changes or subjectivist humanism than to organize the still nascent phenomenological conception of the human being (sometimes called “phenomenological anthropology”) in the form of a scientific corpus. Paradoxically, the rejection of the scientific paradigm hampered any quest to use (phenomenologically informed) scientific methods to design strategies for addressing the epidemiological challenges that the humanistic paradigm might contribute to overcoming. Existentialist phenomenology seems to have had an inherent scepticism toward anything that might be subsumed under technical-administrative principles and thus also institutions. In this, there is a certain conflict between the social need to rebuild societies and the spirit of existentialist deconstructivism, a conflict which contributed to its limited influence at this time.

This is why as we said at the start of this section, the need for both roles of phenomenology is shown in this period by default: the individual-centered role of phenomenology (represented here by existentialist humanism) becomes ineffective when disconnected from the science-centered role of phenomenology. The humanism of the post-war period awakened a new conception of the human being with the aim of influencing the whole of society, something to which phenomenological existentialism contributed indirectly in its twilight years. Yet it became incapable of incorporating all the trends of modern science (new drugs, epidemiological needs, the need for “manualisation” of assessment procedures and therapeutic practices, etc.) which, for better or worse, emerged over this period as an unstoppable wave on the horizon of contemporary mental health care.

## 2. The contemporary period

It is perhaps too early for a comprehensive historical characterization of the contemporary period in mental health–after all, we are still in it! We return to our own “mental health first” take on this in our conclusions. There are, however, insights to be gained into the present period from the perspective on the history of phenomenology outlined in Section I, and it is on these insights that we focus in this section,

As we will indicate, the contemporary period in psychiatry can be understood in terms of what amounts to a progressive uncoupling of academic psychiatry from front-line clinical care. This uncoupling, as we describe below, has two distinct dimensions, respectively science-centered and individual-centered. As such, and guiding our proposal for a new synthesis of phenomenology’s twin roles, the dimensions of this uncoupling correspond broadly with the two historic roles of phenomenology described in Section 1. It is in virtue of this correspondence, we argue toward the end of this section, that phenomenology is uniquely well-placed to respond to the challenges of contemporary mental health. We explore these challenges below in this section, as exemplified by a new form of recovery-oriented practice underpinning person-centered care in contemporary mental health. These challenges, once again, divide naturally into science-centered and individual-centered. Responding, however, to these twin challenges, requires more than a merely twin-track response from phenomenology. It requires what we argue amounts to a dialectical synthesis of phenomenology’s twin historic roles. We describe a number of barriers to, and also resources for, implementation of a synthesis of this kind.

### 2.1. Uncoupling in two dimensions

The uncoupling of academic psychiatry from front-line clinical care, as we have indicated, is in two dimensions, one science-centered, the other individual-centered. The science-centered dimension of this uncoupling is evident in the degree and extent to which papers published in scientific journals can provide information to the clinician that may improve his/her practice. This has been called “clinical factor” to distinguish it from the impact factor (Fava, 2011). An increasing number of researchers who have no or little familiarity with the clinical process and their research products–although published in journals with a high IF–reflect this lack of clinical experience. A consequence of this is the failure of the “new” neurosciences–taking place in this clinical vacuum -the failure of the “new” neurosciences to translate into improvements in front-line clinical care. This failure parallels in a number of respects a corresponding failure of early 20th century psychiatric science. Both periods as we noted at the start of this article, had been preceded by optimistic forecasts: Wilhelm Griesinger’s “psychiatry as brain science” in the late 19th century; the anticipation of the 1990s as the ‘decade of the “brain” in the late 20th century.

In both periods, again, the critics of psychiatric science included those directly involved as academic researchers. In the early 20th century, you will recall from Section I, how Karl Jaspers criticized the scientific *hubris* of his day from the perspective of one who had worked as a neuroscience researcher in Franz Nissl’s brain science laboratory. Similarly today, in the early 21st century, prominent critics of scientific psychiatry have included the authors of the American Psychiatric Association’s latest edition of its diagnostic manual, the DSM 5^1^. Distinguished researchers, David Kupfer, Michael First and Daryl Regier, who later went on to become leaders of the review process leading to DSM 5, pointed to the failure of earlier editions of DSM to generate research leading to improvements in clinical care, arguing that we needed “an as yet unknown paradigm shift” that would “transcend the limitations of the current DSM paradigm” (Kupfer et al., 2002, p xix). Similarly, a few years later, when DSM 5 was eventually published, Thomas Insel, at the time Director of one of the world’s largest neuroscience funding bodies, the USA-based NIMH^2^, felt obliged for much the same reasons (the failures of translation of research into practice) to publish an alternative to DSM for research purposes, the RDoC framework^3^ (Cuthbert and Insel, 2013).

The individual-centered dimension of the uncoupling of academic psychiatry from front-line care, is manifest in a variety of ways, both negative and positive, and both within and beyond psychiatry. Negative manifestations within psychiatry include major issues in fields such as “(…) governance, resources, services, information and technologies for mental health” (World Health Organization, 2022, p.51). Negative manifestations beyond psychiatry, include the exporting of responsibility for mental health care to non-medical disciplines within multi-disciplinary teams. Such teams are important in all areas of contemporary health care but in mental health they reflect a rejection, in part and by some, of the medical model of mental health on which psychiatry is based, including its approach to diagnosis (Kutchins and Kirk, 1997). More radical still, has been a rejection not just of psychiatry as a medical discipline but of professional models of any kind. As a negative aspect of uncoupling, this corresponds with the anti-psychiatry movement of post-War humanism described in Section 1. It has though also a positive counterpart that we believe is unique to the contemporary period.

This positive aspect of the individual-centered dimension of uncoupling, is the growth in importance of expertise-by-experience, not as an alternative to expertise-by-training, but as a complement to it. This is reflected in various aspects of co-production between patients and carers (as experts-by-experience) and professionals of various kinds (as experts-by-training) in both the development (including the development by research) and the delivery of mental health services (Faccio et al., 2023). Clinically, it is reflected in the growing importance of a new understanding of recovery defined, not by the values of (by what matters or is important to) professionals, i.e., paradigmatically, diagnosis and symptom control, but by the values of (by what matters or is important to) *the individual concerned* (see references next section). We describe recovery, so defined, further, and with clinical examples, in the next section. As these examples illustrate, the challenges raised by recovery of this kind are, again, both science-centered and individual-centered. Hence it is that, as we describe in subsequent sections, phenomenology, through a synthesis of its twin historic roles, has a new and potentially important role to play in responding to the challenges of recovery-oriented practice in 21st century mental health care.

### 2.2. Recovery and its twin challenges

Although now increasingly widely adopted within mental health services, contemporary models of recovery were a product of service user-led rather than professional-led initiatives. Developing in parallel in a number of countries, one of the first practical manuals of recovery practice, WRAP^4^, was produced by freelance researcher and author Mary Ellen Copeland based on lived experience (her own and others’) of mental health issues (Copeland, 2005).

Recovery, as defined by Copeland and others, is based on what with hindsight seems a simple idea. Rather than defining recovery by reference to what is important or matters to experts-by-training (such as diagnosis and symptom control), it should be defined by reference to re-establishing a good quality of life as defined by (and this is crucial) the values of (by what is important or matters to) the *individual concerned* (see eg., ; Copeland, 2005; Slade, 2009; Slade et al., 2014).

This might seem impossibly idealistic, particularly in the acute situations so often presented by everyday practice in mental health. Indeed, if someone is unwell, particularly in a crisis situation, they may not be able or want to be actively involved in how their problems are being assessed. The following brief vignette illustrates, however, the vital importance for recovery of understanding what matters to the individual concerned, even, perhaps particularly, in situations of acute care. There is an initial time-cost attached to this. But as the vignette also illustrates, this is an investment that pays for itself in the overall time-cost of care.

#### 2.2.1. Time for Martin

*Martin was admitted to the acute care ward, detained under the Mental Health Act (MHA) and accompanied by police officers and the MHA social worker. Martin suffered from a paranoid delusional disorder which when untreated and acute put others at risk, especially neighbors he believed were entering his property. This had once led to serious physical assault on the teenage daughter of his next-door neighbor after which Martin was admitted to a forensic unit.*

*Martin was threatening and aggressive on admission. The police stayed around for the first half hour and extra nursing staff were in attendance. His anger appeared to have peaked and so the extra security was gradually reduced until there were just two people trying to negotiate with him. Despite Martin’s threats, those concerned felt was that he wanted to talk but was too angry to ask for this.*

*Finally, Martin accepted a cup of tea and sat down with staff. They listened and gave him time and eventually he was able to focus on his two main worries about admission. He feared his water pipes would freeze and then burst and that his house would be flooded. He was concerned too that he would not be able to visit his elderly mother who was in a nursing home close to where he lived.*

*After discussion, Martin went home accompanied by two nurses to make sure his house stayed warm. He returned to the ward visibly settled. The next day, he began to accept medication and within a week, he was having unescorted leave from the ward, visiting his mother and his home and fully engaging with the treatment plan for him. He remained in hospital for approximately another 5 weeks–a significant reduction on previous lengths of stay.*

In this story (which is reproduced here exactly as reported in the original^5^) we see the positive benefits of taking time to understand what is important to the person concerned (Martin’s concerns about his home and his mother). What mattered to those responsible for his care was managing his propensity for violent outbursts (hence the precaution of the two police constables staying for a while). But instead of focusing on this they took time to allow him to settle and gain sufficient confidence to explain what he was worried about. This then became the basis for a successful treatment plan resulting in discharge home after a shorter admission than usual.

To be clear, there are those for whom a diagnosis and symptom control are important to their quality of life (see Colombo et al., 2003; and footnote 7). Such was indeed a component of Martin’s treatment in the above vignette. The aim of recovery practice is thus not to preclude this or that way of intervening with mental health issues but to ensure that interventions are guided–as they were guided in Martin’s story–not by impersonal guidelines alone but by what is important to the individual concerned. This, as Marin’s story again illustrates, is often a “win” for everyone. Having understood and responded to what was important for Martin, staff had no difficulty in getting him to co-operate in medication and other risk management strategies as part of his overall treatment plan.

Recovery, so understood, is both values-based and evidence-based. It is values-based in being defined by individual values, by what is important to the quality of life of the individual concerned. It thus requires input from that individual as an expert-by-experience. But it is also evidence-based, requiring input from one or another expert-by-training on evidence-based ways of achieving (individually-defined) quality of life. Such evidence-based interventions, then, consistently with this model of recovery, may, or (as in Natalie’s case) they may not, include medication for symptom control^6^.

Both aspects of recovery–its evidence-base and its values-base – raise challenges. The evidence base of recovery is challenging, in that, *inter alia*, there is in mental health often no agreed “corpus” of evidence on which to base interventions: professionals with different areas of expertise-by-training (psychiatrists, psychologists, and social workers, for example) may prioritize different approaches to intervention; and, as we have indicated, there are experts-by-experience who reject expertise-by-training of any kind. But the values-base of recovery is no less challenging than its evidence-base. This is essentially because (as we illustrate further below) values in all areas of health care, but especially in mental health, may be both complex and conflicting.

Phenomenology offers a resource for responding to values challenges of both these kinds. In its science-centered role it may contribute to the evidence-base of recovery through any of the ways in which, historically and in the contemporary period, it has contributed to psychiatric science. As one of us has argued elsewhere, there is indeed a sense in which psychopathology should be regarded as the basic science of contemporary psychiatry (Stanghellini and Broome, 2014). A paradigmatic case is that of delusions: despite being described as irrational and implausible beliefs, delusions are meaningful, they may also enhance the sense that one’s life is meaningful, supporting agency and creativity in some circumstances, they can help make sense of one’s unusual experiences and in some circumstances even support one’s endeavors, albeit temporarily and imperfectly. Acknowledging that delusions have meaning and can also give meaning to people’s lives has implications for our understanding of psychotic symptoms and for addressing the stigma associated with psychiatric conditions (Parnas and Sass, 2001; Stanghellini and Raballo, 2015; Ritunnano et al., 2021; Ritunnano and Bortolotti, 2022). We return below to further examples from the contemporary period. In its individual-centered role phenomenology may contribute in various ways to the values-base of recovery. In the next section we give a very brief introduction to values-based practice and then illustrate two ways in which phenomenology is already turbocharging the resources of values-based practice for responding to the particular values challenges presented by mental health.

### 2.3. Values-based practice and phenomenology

Values-based practice is a relatively recent addition to the resources of contemporary health care for working with complex and conflicting values (Fulford et al., 2012). Earlier established resources include ethics and law. Values-based practice is distinctive in focusing on individual values. In this, as its name implies, it is closer to, and indeed in clinical contexts (including that of recovery) it works best as a partner to, evidence-based practice. This partnership reflects the fact that they both involve processes that support decision-making in health care: rather than giving pre-set answers, both rely on processes to support those concerned in coming to decisions for themselves according to the particular circumstances presented by the situation in question. Evidence-based practice provides a support process (based on meta-analyses of high-quality research and a consensual model of decision-making) for decisions where complex and conflicting *evidence* is involved. Values-based practice provides a support process (based on learnable clinical skills and a “dissensual” model of decision-making^7^) for decisions where complex and conflicting *values* are involved.

Values-based practice, although developed first in mental health, offers a resource for all areas of health care. The current Director of the Centre for Values-based Practice in Oxford is a vascular surgeon, Oxford’s Tutor for Surgery, Professor Ashok Handa; and much recent development of the field has been in areas of bodily medicine including, besides surgery, radiology and emergency care. But mental health, too, has seen active development in recent years. These developments, returning to the challenges of recovery-oriented practice, reflect in particular some of the many ways in which phenomenology, building on the origins of values-base practice in “ordinary language” analytic philosophy (Fulford and van Staden, 2013), adds to its resources for responding to the particular values challenges of mental health practice (Fulford and Stanghellini, 2019; Messas and Fulford, 2021a,b).

We are aware of course of the equivocal relationship between phenomenology and values. Our view on the patients’ values is covered by the inclusive model underpinning values-based practice. The latter model, indeed, we have argued elsewhere, maps directly on to the phenomenological concept of the patient’s attitude to his or her disorder (Stanghellini et al., 2013). Be that as it may, the following examples illustrate the impact of two distinct areas of phenomenology on the particular values challenges presented by mental health, respectively, the insights to be gained from Sartre’s three-way body phenomenology into the empathically obscure values operative in anorexia, and the interpretive and communicative roles of dialectical phenomenology in working with conflicting values arising in addictive disorders^8^.

#### 2.3.1. Sartre’s body phenomenology and empathically obscure values in anorexia

*Ana (not her real name) was in her twenties when she was referred to mental health services at the request of her family, with progressive weight loss. Consistently with the DSM criteria for anorexia nervosa, she showed: (A) significantly low body weight for her age due to “restriction of energy intake”; (B) “Intense fear of gaining weight or of becoming fat”; and (C) “Disturbance in the way (she experienced her) body weight or shape, undue influence of body weight or shape on self-evaluation, or persistent lack of recognition of the seriousness of (her) current low body weight.”*

*Her weight loss was highly important in Ana’s scale of values. In DSM terms: her “self-esteem*… *(was) highly dependent on (her) perceptions of body weight and shape”; and her “weight loss (was)*… *viewed (by her) as an impressive achievement and a sign of extraordinary self-discipline, whereas weight gain (was) perceived as an unacceptable failure of self-control.”*

*Ana had a troubled first-person perspective on her body, describing this as, “I don’t feel my body. It changes in shape and consistency according to situations”; again, it’s like a “wobbling liquid whose shape changes when I’m in the presence of other people.” She expressed shame and disgust in relation to her body, while, at the same time, seeking identity through the others’ gaze: for example, when a group of young men looked at her as she passed them in the street, “I realized that I had a sensation of ‘unity’ when I felt they were watching me. It was as if their looks were ‘condensing’ me. I ‘focalize’ myself through their gazes.”*

Ana’s values as described in this story, although characteristic of people with this condition^9^, are for most people empathically obscure. Complexity is a feature of health-related values in all areas of healthcare: head-line ethical values, for example, such as “beneficence” and “autonomy” are complex in the sense that their meanings vary from situation to situation, from person to person, and so forth. This is why training in values-based practice includes raising awareness of the diversity of meanings attached even to ostensibly shared values (Fulford et al., 2012, chapter 4). But in conditions such as anorexia an additional level of complexity is added by the values in question being not only different but deeply difficult to understand. For Ana, weight loss is at the center of her self-esteem: so much so, that she is at risk of dying. But whereas non-voice hearers can understand (if not share) the importance Martin placed on his house suffering water damage or his mother feeling abandoned in a “home,” non-anorexics (including in Ana’s story, members of her family) find the value she places on low body weight (even at the cost of dying) entirely obscure.

This is where, as one of us has shown elsewhere (Stanghellini, 2017), phenomenological insights are potentially illuminating. Drawing in this instance on Jean Paul Sartre’s three-way phenomenology of the body (Sartre, 1986), the values underpinning anorexia can be shown to reflect an unbalanced influence of the body as perceived by other people. The importance of the other’s gaze is reflected in Ana’s story for example in the contrast between her first-personal and third-personal experiences of her body: her “troubled” first personal experiences are steadied by her third-personal experience of being looked at by a group of young men in the street.

We do not have space here to set out this explanation in detail:^10^ Stanghellini (2019) has developed it elsewhere both as the basis of a clinical intervention (Stanghellini and Mancini, 2017) and in a combined empirical-phenomenological research paradigm (Stanghellini et al., 2012). As such, this work offers a powerful example of the resources potentially available from phenomenology for responding to complexity of values in mental health. Our second case offers an example of the corresponding role of phenomenology for responding to conflicting values in mental health^11,12^.

#### 2.3.2. Dialectical phenomenology and conflicting values in addiction

*Bruno (not his real name) was a 50-year-old patient receiving treatment for Alcohol Use Disorder following an acute episode of alcoholic hepatitis, for which he had been hospitalized. He was regular and punctual in his follow up appointments, attending both psychiatric and hepatology clinics accompanied by his wife, Lucia (also not her real name)*.

*Bruno had been very frightened by the impact of alcohol consumption on his health and after a lifetime of drinking, he had decided to stop. But at his next appointment, with psychiatrist, Dr. Sousa (not her real name), he admitted he no longer wished to give up his drinking. Yes, he was aware of the dangers, both to his health (one of his uncles died of liver cirrhosis), and to his work. Furthermore, his wife, Lucia, on whom he said he relied heavily (“she is the only one I can trust”), had threatened to leave him unless he stopped drinking. But for all this, he believed he was strong enough to manage what he (though no one else in his family) regarded as his relatively modest continued consumption of alcohol; and he valued this (and the social life at his work that went with it) ahead of the risks.*

*Bruno refused treatment with disulfiram (because he knew this would make drinking impossible for him); he also rejected the idea of joining a group to support abstinence (because, as he said again, he did not wish to be abstinent!). He was, however, keen for his outpatient appointments to continue and for his physical health to be monitored. One day, he acknowledged, he would have to stop drinking, but “not now.”*

*The policy of the clinic was to refuse follow up unless a patient had agreed to become abstinent, but Dr. Sousa, as the doctor directly involved, believed Bruno and Lucia should be offered on-going support.*

There are clearly a number of conflicting values already in play in this story: between Bruno and his wife, Lucia (and in the fuller version of this story with other members of Bruno’s family as well); between Bruno and the clinicians involved in running the clinic; and between Dr. Sousa and these same clinicians (over whether or not Bruno should be offered treatment). For purposes of resolving conflicts of these kinds, in which the values in question are relatively transparent, the generic resources of values-based practice will normally be sufficient: these include, centrally, communication skills such as listening, “giving space,” and so forth^13^.

Dr. Sousa demonstrated these skills successfully in the context of this story. But her advocacy of continued support was driven by her understanding of a further clinically-relevant conflict of values, a conflict involving values that were very far from being transparent, and for which in consequence the generic skills of values-based practice would not have been sufficient. This further conflict was a conflict *within Bruno himself*. Bruno, like many others with addictive disorders, both wanted, and did not want, to give up his addiction. On the one hand, he was refusing treatment designed to help him become abstinent. But on the other hand, he showed awareness of the risks he was running and wanted to continue his contact with the clinic.

Dr. Sousa had supplemented her medical training as a psychiatrist by taking a course in phenomenological psychopathology; and as a specialist in addictive disorders, she had been impressed with the insights to be gained particularly from dialectical phenomenology (Messas, 2021). Dialectical phenomenology has a number of features in common with values-based practice–its focus, in particular, not on individual elements of the pre-reflective life world but on the *balance between pairs* of such elements, parallels the balancing of values that is at the heart of values-based practice. The pairs concerned furthermore include values explicitly; and, importantly for recovery practice, they include values expressing strengths as well as needs and difficulties (for further discussion, see Messas and Fulford, 2021a,b).

Thus informed by dialectical phenomenology, Dr. Sousa understood Bruno’s life world as being in an imbalance of “personal” as against “interpersonal” elements and of “present” as against “future” elements. Add to this, the important strength in his situation represented by his wife, Lucia’s, support, and it was a small step to Dr. Sousa’s decision to offer Bruno and Lucia on-going support notwithstanding the policy of the clinic. It was through such support, she believed, based on her insights from dialectical phenomenology, that Bruno was most likely to find a way of rebalancing the elements of his life world, building up the importance of future considerations as against the present, and of his concerns for his relationships with others (at work as well as in his family) as well for his own gratification. Consistently, however, with the importance of combining values-based with evidence-based approaches, Dr. Sousa supplemented her phenomenologically-informed follow up strategy with topiramate (recommended for reducing the urge to drink alcohol) and naltrexone (recommended for reducing the pleasure obtained from drinking alcohol). In contrast to his rejection of her earlier suggestions, Bruno accepted a trial of both of these medications in the context of his on-going access to care.

### 2.4. A proposed dialectical synthesis of roles

The contemporary period in psychiatry, as we noted at the start of this article, has been marked, as the early 20th century was marked, by a turn to phenomenology largely in response to perceived failures of psychiatric science. In the present period, for example, Andreasen (2007) pointed to the “death” of psychopathology as an “unintended consequence” of the shift to operationalism in DSM; and a number of leading psychiatric journals have carried articles on phenomenological themes, including Lancet Psychiatry (2021) (for example, a 2021 editorial, “The things themselves” and related articles; also Messas and Fulford, 2021a) and *World Psychiatry* (for example, its 2015 Forum on Phenomenological and neuroscientific perspectives on delusion; lead article, Sass and Byrom, 2015, and a series of articles including, e.g., Fusar-Poli et al., 2022).

Thus far it would seem, what we have identified as the science-centered role of phenomenology has been, as it was in Jaspers’ work, to the fore. There is an important difference, however, in that as we also noted above, the individual-centered role of phenomenology (reflected in the rise of expertise-by-experience with its associated recovery-oriented practice) has been explicitly (and not merely implicitly) in evidence as well. Thus, Jaspers’ work, as we described in Section I, although explicitly science-centered, drew implicitly on the individual-centered role of phenomenology in his reliance on the resources of the “human sciences” for establishing meaningful connections alongside the causal connections established by the empirical sciences: the result, as we described, was the phenomenology of *General Psychopathology*. The two roles, we went on the suggest, were balanced the other way in the structural phenomenology that developed a few years later: although notable for a “new” anthropology, the “structuralists,” as Elizabeth Basso (cited above) argued, remained focused on key aspects of the agenda of the empirical sciences in psychiatry, such as diagnosis.

Contemporary phenomenology, by contrast, exhibits elements of both roles, not only in equal measure but, with many authors, working together in parallel. Other authors, moreover, while adopting overtly individual-centered approaches, remain science-centered as well: for example, Sass (2017) has drawn deeply on the arts and literature in redescribing psychotic conditions; and Matthew Broome, although widely published in the neurosciences, runs (with Giovanni Stanghellini) a flag-ship research program in the UK, *Renewing Phenomenological Psychopathology*, that is overtly individual-centered in that the “renewal” it seeks is by way of enriching phenomenology with resources from the humanities.^14^

That the two roles of phenomenology are in the context of today’s mental health care, being deployed together, is to be welcomed. As we saw in Section 1, the two roles are not readily separable; and an exclusive approach, where it has been tried (as in the period of post-War humanism) has been, ultimately, unsuccessful. Contemporary “parallel-role” work in phenomenology, by contrast, as the examples noted above indicate, is becoming increasingly recognized in mainstream psychiatry.

For purposes of traditional science-based psychiatry this may be sufficient. Our view though is that we need to go further if phenomenology is to support the recovery-oriented practice underpinning contemporary person-centered mental health care. Our view, in essence, is that an effective phenomenological response to what we have described above as the double challenges of recovery-oriented practice, requires, not merely a combination but a synthesis of its two roles. This is essentially because, we suggest, what we have described as the “double” challenges of recovery-oriented practice are in reality two sides of the same (conceptual) coin, namely, the concept of a “person.”

This suggestion builds on a proposal by the British analytic philosopher, Peter Strawson, that the concept of a “person” uniquely and (logically) irreducibly supports ascriptions of both conscious states and bodily states–as Strawson put it, “the concept of a person is the concept of a type of entity, such that both predicates ascribing states of consciousness and predicates ascribing corporeal characteristics, a physical situation & co. are equally applicable to a single individual of that single type” (Strawson, 1977, pps, 101, 102). We do not have space to develop this idea in detail. A full account even of its theoretical basis, besides engaging with the philosophical literature generated by Strawson’s account of “person,” would require, as a minimum, a review of the diversity of meanings attached to the notion of “person-centered practice” in contemporary health care (Fulford, 2020). Added to which, if it is to be taken seriously as a practical proposal, are all the generic challenges of professional identity, resources, and so forth, that stand in the way of admitting “expertise-by-experience” alongside established “expertise-by-training.” Psychiatry is also massively influenced by how funders (whether private health insurers or national providers) limit provision. This is mostly to limit expenses and avoid social conflicts, rather than maximize recovery outcomes. Is psychiatry as practiced stuck because it suits funders to stick to symptom driven (and then prescription driven) ways of conducting the work? How could phenomenological psychiatry “speak to” the concerns to this form of power and its underlying epistemology? It is not the purpose of this paper to address such challenges, but we should not underestimate their impact: the extent to which service provision is driven by costs and social control will be all too evident equally to those with expertise-by-experience and expertise-by-training.

Easy to state, then, such a synthesis faces many barriers. Besides, the metaphysical challenges (not least as just noted concerning the nature of “persons”), practical barriers include professional, organizational and educational factors. We noted in Section 1 some of the challenges of access faced by the structural phenomenologists. Indeed, the wider lesson of history itself, is that as the North American humourist, Mencken (1920), put it, “For every complex human problem, there is always at least one solution that is simple, neat … and wrong.” The very framing of the issues in this paper – in effect divided between the “sciences” and “humanities”–reflects the attractions of the simplifications of which Mencken’s aphorism warns.

There are, it is true, new resources supporting implementation as well. In addition to all the resources of the expanding field of contemporary phenomenological psychopathology (Stanghellini et al., 2019), there are resources of theory from other areas of philosophy: from analytic philosophy (on the concept of “person,” as above); and on the relational nature of values (see, e.g., Bergqvist, 2018): and from non-European philosophies (such as Batho Pele, a distinct form of values-based practice based on African philosophical concepts that bridge the individual/social divide, van Staden, 2021). While as to practical resources, co-production between experts-by-training and experts-by-experience, for all the challenges it presents to traditional models of professionalism, is a potentially powerful resource supporting developments in person-centered practice in all areas of health care.

But for all the resources, there is no doubting the difficulties facing implementation. Which is why, recognizing the current state of development of the field, our proposal is not, as such, for a synthesis of phenomenology’s twin roles, but rather for a *dialectical* synthesis. Our proposal is that rather than working with phenomenology’s twin roles in parallel, still less seeking premature closure by way of Mencken-style simplifications, we should engage with them in a dialectic aimed, not at resolving the differences between them, but at continually sharpening them up and bringing them back to the center of our attention. Thus, to finish with a conjecture, may today’s dialectic between yesterday’s twin roles of phenomenology emerge as tomorrow’s new synthesis supporting recovery-oriented mental health care.

## 3. Conclusion—mental health first

In this paper we have drawn on the lessons of history to argue that a dialectical synthesis of phenomenology’s traditionally twin roles in psychiatry, would make it uniquely well-equipped to support the model of recovery-oriented practice underpinning contemporary person-centered mental health care. We identify the twin roles of phenomenology in psychiatry, respectively as science-centered (with phenomenology focused on enhancing the role of empirical science in psychiatry) and individual-centered (with phenomenology focused on enhancing the role of humanism in psychiatry). Section I of the paper illustrates how these roles play out separately in different ways and to different degrees as response to different challenges faced by psychiatry during three exemplar periods of the 20th century: during Karl Jaspers’ introduction of phenomenology to psychiatry in his 1913 *General Psychopathology*; during the development a few years later of structural phenomenological psychopathology; and in response to the rise of post-World-War II humanism.

Section II of the paper then turns to the contemporary scene. This has witnessed a new “turn to phenomenology” in which, unlike earlier periods, its two roles often appear working side-by-side. This reflects a contemporary challenge that we describe as a progressive uncoupling of academic psychiatry from front-line clinical care, an uncoupling that, corresponding with the two roles of phenomenology, presents both science-centered and individual-centered aspects. The science-centered aspect of the uncoupling is reflected in a failure of translation of the “new” neurosciences into improvements in clinical care. Its individual-centered aspect is reflected in the rise of “expertise-by-experience” standing alongside and in a co-productive relationship with traditional “expertise-by-training.” A key product of such co-production, unique to the contemporary period, is a model of “recovery” that is defined, not by the values of (by what is important or matters to) professionals as experts-by-training (such as diagnosis and symptom control), but by the values of (by what is important or matters to) patients and carers as experts-by-experience.

We illustrated the significance of recovery, so defined, with a brief clinical vignette (of hearing voices). As such, we noted, recovery raises acute challenges for both the evidence-base and the values-base of clinical decision-making. It is as a response these challenges, we argued, that phenomenology, in virtue of its twin roles, offers a unique resource. Its potential role in respect of the evidence-base of recovery, is reflected, we suggested, in its growing recognition as the “science” of contemporary clinical care. Its potential role in respect of the values-base of recovery is less well recognized; but we illustrated it with two further clinical examples, respectively of anorexia (drawing on Sartre’s three-way phenomenology of the body) and of addiction (drawing on dialectical phenomenology).

These examples, taken separately, provide a degree of proof of principle of the potential of phenomenology in supporting recovery. But only a synthesis of its roles, we then argued, would make it fully effective in this respect. Noting the many difficulties, practical and theoretical, to a synthesis of phenomenology’s twin roles of the kind we propose, and warning against premature closure by way of unwarranted simplifications on these difficulties, we suggested instead that in the present state of development of the field, we should aim rather for a *dialectical* synthesis of phenomenology’s twin roles. This prompted our concluding conjecture, that it is from such a dialectic that a genuine synthesis of roles may ultimately emerge.

We will finish on a “mental health first” historical note. The position of psychiatry in 20th century scientific medicine was widely characterized as that of a running in second place to more “high tech” areas of health care. The importance of phenomenology in psychiatry, and its potential role in respect particularly of the values-base of recovery, may appear to endorse this “psychiatry second” stereotype. But this is entirely wrong. As one of us has argued elsewhere (Fulford, 1989), the essentially value-laden nature of the medical concepts, means that advances in medical science and technology increase the importance not only of the evidence-base of clinical decision-making but also of its values-base. This is essentially because such advances open up new choices for patients and with choices go values. If this is right, then psychiatry, in drawing on phenomenology to develop the resources for meeting the challenges of recovery-oriented practice, is not running second to, but actually leading the way, in developing the resources for person-centered practice for health care as a whole.

## Data availability statement

The original contributions presented in this study are included in the article/supplementary material, further inquiries can be directed to the corresponding author.

## Author contributions

All authors listed have made a substantial, direct, and intellectual contribution to the work, and approved it for publication.

## Footnotes

1 The fifth edition of the Diagnostic and Statistical Manual (American Psychiatric Association, 2022).

2 National Institute for Mental Health.

3 RDoC stands for Research Domain Criteria.

4 The Wellness Recovery Action Plan (Copeland, 2005).

5 Martin’s story is described among other examples of good practice in mental health assessment in the *3 Keys to a Shared Approach in Mental Health Assessment* [The National Institute for Mental Health in England (NIMHE) and the Care Services Improvement Partnership, 2008]. The *3 Keys* was one of a series of guidance documents on values- and evidence-based mental health practice produced by the UK Department of Health based on wide-raging consultations with stakeholders including both experts-by-experience (patients and carers) and experts-by-training (psychiatrists, mental health nurses, psychologists and others).

6 In developing her WRAP model (above this section), Mary Ellen Copeland was among those who at the time were seeking approaches to managing mental health issues other than through medication and similar “medical” means. There is though evidence that service users are split between those who value medical interventions and those who do not (see, e.g., Colombo et al., 2003). Hence by its own principles, the recovery model accommodates approaches of both kinds.

7 For more on values-based practice, see the website of the Collaborating Centre for Values-based Practice in Health and Social Care, at St Catherine’s College, Oxford: https://valuesbasedpractice.org. Among other resources, this includes a detailed reading guide and free-to-use down-loadable resources.

8 Ana’s story is an amalgam of cases from the extensive work on feeding and eating disorders carried out by one of us and partially included in Stanghellini (2017); for more on values-based practice, phenomenology and values, see Stanghellini and Fulford (2020).

9 Besides their reflection in DSM, similar values are at the heart of “pro-Ana” and “Pro-ed” (Eating Disorder) websites promoting anorexia and related conditions as a way of life.

10 For further discussion, see Fulford and Stanghellini (2019) and Stanghellini and Fulford (2020).

11 Based on Messas and Soares (2021) – this gives a longer version of Bruno and Lucia’s story; see also, Messas and Fulford (2021a).

12 This medication induces feelings of nausea when the patient drinks alcohol.

13 For further details, see e.g., Fulford and Benington, 2004; Fulford et al., 2012, especially, chapters 7 and 14.

14
https://www.birmingham.ac.uk/research/mental-health/renewing-phenomenological-psychopathology/about-the-project.aspx
